# Intraoperative mapping of the right hemisphere: a systematic review of protocols that evaluate cognitive and social cognitive functions

**DOI:** 10.3389/fpsyg.2024.1415523

**Published:** 2024-06-20

**Authors:** Isabel Martín-Monzón, Laura Amores-Carrera, David Sabsevitz, Guillaume Herbet

**Affiliations:** ^1^Department of Experimental Psychology, Faculty of Psychology, Campus Santiago Ramón y Cajal, University of Seville, Seville, Spain; ^2^Department of Psychiatry and Psychology, Division of Neuropsychology, Mayo Clinic Florida, Jacksonville, FL, United States; ^3^Department of Neurosurgery, Gui de Chauliac Hospital, Montpellier, France; ^4^Praxiling Lab, UMR5267 CNRS & Paul Valéry University, Bâtiment de Recherche Marc Bloch, Montpellier, France; ^5^Department of Medicine, University of Montpellier, Campus ADV, Montpellier, France; ^6^Institut Universitaire de France, Paris, France

**Keywords:** right hemisphere, awake surgery, direct electrical stimulation, cognitive mapping, neuropsychological protocol, intraoperative brain mapping, connectome

## Abstract

The right hemisphere of the brain is often referred to as the non-dominant hemisphere. Though this is meant to highlight the specialized role of the left hemisphere in language, the use of this term runs the risk of oversimplifying or minimizing the essential functions of the right hemisphere. There is accumulating evidence from functional MRI, clinical lesion studies, and intraoperative mapping data that implicate the right hemisphere in a diverse array of cognitive functions, including visuospatial functions, attentional processes, and social cognitive functions. Neuropsychological deficits following right hemisphere resections are well-documented, but there is a general paucity of literature focusing on how to best map these functions during awake brain surgery to minimize such deficits. To address this gap in the literature, a systematic review was conducted to examine the cognitive and emotional processes associated with the right hemisphere and the neuropsychological tasks frequently used for mapping the right hemisphere during awake brain tumor surgery. It was found that the most employed tests to assess language and speech functions in patients with lesions in the right cerebral hemisphere were the naming task and the Pyramids and Palm Trees Test (PPTT). Spatial cognition was typically evaluated using the line bisection task, while social cognition was assessed through the Reading the Mind in the Eyes (RME) test. Dual-tasking and the movement of the upper and lower limbs were the most frequently used methods to evaluate motor/sensory functions. Executive functions were typically assessed using the N-back test and Stroop test. To the best of our knowledge, this is the first comprehensive review to help provide guidance on the cognitive functions most at risk and methods to map such functions during right awake brain surgery.

**Systematic Review Registration:**

PROSPERO database [CRD42023483324].

## 1 Introduction

Direct electrical stimulation (DES) during awake craniotomy for brain tumors has contributed substantially to our understanding of functional neuroanatomy. Nevertheless, most of this work has focused on the dominant hemisphere or left hemisphere functions, mainly speech and language; the right hemisphere, typically referred to as the non-dominant hemisphere, is often considered less eloquent than the left hemisphere, especially in clinical fields. This is highlighted by the preference in many neurosurgical centers to perform left hemisphere resections awake with DES, while right hemisphere resections not involving the sensorimotor cortex are often done under general anesthesia. The view that the right hemisphere is less eloquent than the left hemisphere is challenged by the many lesion and neuroimaging studies (Bartolomeo, 2006; Gianotti, 2012; Robertson, 2014) showing that a variety of functions depend on the integrity of the right hemisphere, including attention, visuospatial, emotional, and social cognitive functions. Further, high rates of cognitive morbidity have been reported after right hemisphere resections (Vilasboas et al., 2017).

Herbet and Duffau (2020) highlighted the critical importance of mapping the right hemisphere as well as the cortico-subcortical distributed networks mediating movement execution and control, visual and spatial cognition, language processing, working memory, and social cognition. This is particularly noteworthy given the observed constraints in neuroplasticity potential within these connections (Herbet et al., 2016). Following their work, different teams around the world have started to introduce new tasks to monitor a variety of cognitive functions, including sensorimotor, language, attention, visuospatial, emotional, and social functions. Duffau and his group have made significant contributions to the literature by extending mapping to the white matter pathways (Duffau et al., 2002) and, in collaboration with neuropsychologists, introduced new cognitive tasks to the operating room, such as the line bisection paradigm (Thiebaut de Schotten et al., 2005).

Intraoperative stimulation mapping is critical in optimizing the extent of resection while sparing cognitive functions and improving patients' quality of life in both left and right hemisphere surgeries (De Witt Hamer et al., 2012; Wijnenga et al., 2018). Areas usually thought of as “noneloquent” can be essential for brain functions, such as the crucial role of both the right inferior frontal gyrus and the right dorsolateral prefrontal cortex in mentalizing (Yordanova et al., 2017), the right dorsolateral prefrontal cortex in nonverbal semantic processing (Herbet et al., 2018), the right fronto-temporo-parietal cortex in attention (Krall et al., 2015), or right-sided speech functions (Vilasboas et al., 2017).

There is little research to guide the selection of tasks to optimally map these functions with DES. To help inform task selection, it is important to summarize the neuropsychological tasks frequently used for mapping the right hemisphere during awake brain tumor surgery and their correlates with neuroanatomy based on a network model. This study seeks to provide a systematic review of the literature on intraoperative protocols used to map the right hemisphere during awake surgery.

## 2 Methods

Details of the protocol for this systematic review were registered in the PROSPERO database (CRD42023483324).

### 2.1 Search strategy

A systematic literature review was conducted following the PRISMA guidelines (Preferred Reporting Items for Systematic Reviews and Meta-Analyzes) in order to adjust the review methodology to quality criteria (Liberti et al., 2009; Moher et al., 2009). The databases used to search for articles were PubMed, PsycINFO, Scopus, and Cochrane Library. All of these databases were chosen because they contain articles related to neuroscience in general (PubMed, Scopus, Cochrane Library) and specifically psychology (PsycINFO).

The search process was carried out systematically in these databases, with words and Boolean terms: (“awake brain surgery” OR “awake craniotomy” OR “awake brain mapping” OR “intraoperative mapping”) AND (“right hemisphere” OR “non-dominant hemisphere”) AND (“cognitive functions” OR “cognitive processes”) AND “social cognition” in different combinations. The results were limited to articles published in academic journals in the last 19 years (2005–2024) because it is a recent study topic, especially in the field of neurosurgery. The search in the databases was carried out up to March 2024, obtaining a total of 138 articles in PubMed, 110 in Scopus, 102 in PsycINFO, and 99 in Cochrane Library.

**Figure 1:**
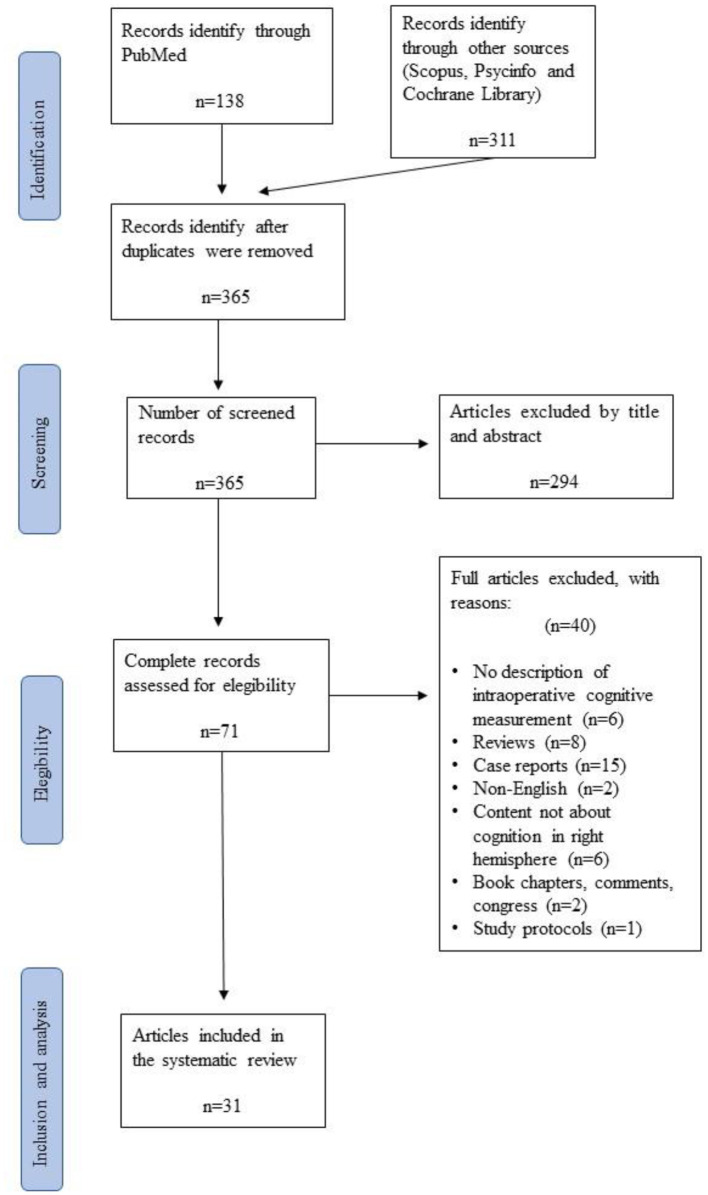
Preferred reporting items for systematic reviews and meta-analyses (PRISMA) flowchart illustrating the search, screening, inclusion, and exclusion process for the present study.

After the first screening, 84 duplicate articles were removed, and a total of 294 were excluded by title and abstract to ensure the selection of relevant articles for further evaluation. Each article was carefully reviewed based on the following criteria: relevance to the research topic, clarity of reported outcomes, methodological alignment, and quality of evidence. Specifically, articles that did not directly address awake craniotomy with direct electrical stimulation in adult glioma patients were excluded. Additionally, articles with unclear or ambiguous reported outcomes related to cognitive functions during awake brain surgery were also excluded. Furthermore, articles that did not align with the methodological framework of our study, such as those assessing cognitive processes not pertinent to right hemisphere surgeries, were excluded. The second phase of screening consisted of an exhaustive reading of the 71 full articles in order to check in Section 2 for the most important variables related to our research question: the number of subjects studied, their age, the surgery procedure followed, the pathologies of the participants, and the neuropsychological instruments informed. This first analysis revealed that some articles did not meet the eligibility criteria, and 40 publications were finally discarded due to the omission of relevant data points. Figure 1 shows the complete study selection process, which ended with 31 articles finally chosen for the systematic review. Authors, titles, year, and code per publication are specified in Table 1.

**Table 1:** Articles considered by code, authors, year and title included in the systematic review.

**Cod**.	**Authors**	**Year**	**Title**	**References**
1	Tomasino, B., Gurracino, I., Ius, T., and Skrap, M.	2023	Continuous real-time neuropsychological testing during the resection phase in left and right prefrontal brain tumors	Tomasino et al., 2023
2	Hartung, S.L., Mandonnet, E., Witt Hamer, P., Klein, M., Wager, M., Rech F. et al	2021	Impaired set-shifting from dorsal stream disconnection: insights from a European series of right parietal lower-grade glioma resection	Hartung et al., 2021
3	Nakada, M., Nakajima, R., Okita, H., Nakade, Y., Yuno, T. Tanaka, S., and Kinoshita, M.	2021	Awake surgery for right frontal lobe glioma can preserve visuospatial cognition and spatial working memory	Nakada et al., 2021
4	Nakajima, R., Kinoshita, M., Okita, H., Liu, Z., and Nakada, M.	2021	Preserving the right pre-motor and posterior prefrontal cortices contributes to maintaining overall basic emotion	Nakajima et al., 2021
5	Prat-Acín, R., Galeano-Senabre, I., López-Ruíz, P., Ayuso-Sacido, A., and Espert-Tortajada, R.	2021	Intraoperative brain mapping of language, cognitive functions, and social cognition in awake surgery of low-grade gliomas located in the right non-dominant hemisphere	Prat-Acín et al., 2021
6	Roux, A., Lemaitre, A.L., Deverdun, J., Ng, S., Duffau, D., and Herbet, G.	2021	Combining electrostimulation with fiber tracking to stratify the inferior fronto-occipital fasciculus	Roux et al., 2021
7	Sarubbo, S., Tate, M., De Benedictis, A., Merler, S., Moritz-Gasser, S., Herbet, G., and Duffau, H.	2020	Mapping critical cortical hubs and white matter pathways by direct electrical stimulation: an original functional atlas of the human brain	Sarubbo et al., 2020
8	Puglisi, G., Howells, H., Sciortino, T., Leonetti, A., Rossi, M., Conti Nibali, M. et al.	2019	Frontal pathways in cognitive control: direct evidence from intraoperative stimulation and diffusion tractography	Puglisi et al., 2019
9	Rech, F., Herbet, G., Gaudeau, Y., Mézières, S., Moureau, J.M., Moritz-Gasser, S., and Duffau, H.	2019	A probabilistic map of negative motor areas of the upper limb and face: a brain stimulation study	Rech et al., 2019
10	Herbet, G., Moritz-Gasser, S., and Duffau, H.	2018	Electrical stimulation of the dorsolateral prefrontal cortex impairs semantic cognition	Herbet et al., 2018
11	Motomura, K., Chalise, L., Ohka, F., Aoki, K., Tanahashi, K., Hirano, M. et al	2018	Supratotal resection of diffuse frontal lower-grade gliomas with awake brain mapping, preserving motor, language, and neurocognitive functions	Motomura et al., 2018
12	Nakajima, R., Kinoshita, M., Okita, H., Yahata, T., Matsui, M., and Nakada, M.	2018	Neural networks mediating high-level mentalizing in patients with right cerebral hemispheric gliomas	Nakajima et al., 2018a
13	Nakajima, R., Yordanova, Y.N., Duffau, H., and Herbet, G.	2018	Neuropsychological evidence for the crucial role of the right arcuate fasciculus in the face-based mentalizing network: A disconnection analysis	Nakajima et al., 2018b
14	Puglisi, G., Sciortino, T., Rossi, M., Leonetti, A., Fornia, L., Conti Nibali, M. et al	2018	Preserving executive functions in non-dominant frontal lobe glioma surgery: an intraoperative tool	Puglisi et al., 2018
15	Rolland, A., Herbet, G., and Duffau, H.	2018	Awake surgery for gliomas within the right inferior parietal lobule: new insights into the functional connectivity gained from stimulation mapping and surgical implications	Rolland et al., 2018
16	Rossi, M., Fornia, L., Puglisi, G., Leonetti, A., Zuccon, G., Fava, E. et al	2018	Assessment of the praxis circuit in glioma surgery to reduce the incidence of post-operative and long-term apraxia: a new intraoperative test	Rossi et al., 2018
17	Zacà, D., Corsini, F., Rozzanigo, U., Dallabona, M., Avesani, P., Annichiaricco, L. et al	2018	Whole-brain network connectivity underlying human speech articulation as emerged, integrating direct electric stimulation, resting state fMRI, and tractography	Zacà et al., 2018
18	Herbet, G., Moritz-Gasser, S., and Duffau, H.	2017	Direct evidence for the contributive role of the right inferior fronto-occipital fasciculus in non-verbal semantic cognition	Herbet et al., 2017
19	Nakajima, R., Kinoshita, M., Miyashita, K., Okita, H., Genda, R., Yahata, T. et al	2017	Damage of the right dorsal superior longitudinal fascicle by awake surgery for glioma causes persistent visuospatial dysfunction	Nakajima et al., 2017
20	Yordanova, Y.N., Duffau, H., and Herbet, G.	2017	Neural pathways subserving face-based mentalizing	Yordanova et al., 2017
21	Rech, F., Herbet, G., Moritz-Gasser, S., and Duffau, H.	2016	Somatotopic organization of the white matter tracts underpinning motor control in humans: an electrical stimulation study	Rech et al., 2016
22	Charras, P., Herbet, G., Deverdun, J., Champfleur, N.M., Duffau, H., Bartolomeo, P. et al	2014	Functional reorganization of the attentional networks in low-grade glioma patients: a longitudinal study	Charras et al., 2015
23	Herbet, G., Lafargue, G., Bonnetblanc, F., Moritz-Gasser, S., Menjot de Champfleur, N., and Duffau, H.	2014	Inferring a dual-stream model of mentalizing from associative white matter fibers disconnection	Herbet et al., 2014
24	Kinoshita, M., Menjot de Champfleur, N., Deverdun, J., Herbet, G., and Duffau, H.	2014	Role of the fronto-striatal tract and frontal aslant tract in movement and speech: an axonal mapping study	Kinoshita et al., 2014
25	Roux, F.E., Dufor, O., Lauwers-Cances, V., Boukhatem, L., Brauge, D., Draper, L. et al	2011	Electrostimulation mapping of spatial neglect	Roux et al., 2011
26	Tate, M.C., Herbet, G., Moritz-Gasser, S., Tate, J.E., and Duffau, H.	2014	Probabilistic map of critical functional regions of the human cerebral cortex: Broca's area revisited	Tate et al., 2014
27	Giussani, C., Pirillo, D., and Roux, F.E.	2010	Mirror of the soul: a cortical stimulation study on recognition of facial emotions	Giussani et al., 2010
28	Shinoura, N., Yoshida, M., Yamada, R., Tabei, Y., Saito, K., Suzuki, Y., and Yagi, K.	2010	Combined damage to the right hemispheric hand area in the primary motor and sensory area plays a critical role in motor hemineglect	Shinoura et al., 2010
29	Vassal, M., Le Bars, E., Moritz-Gasser, S., Menjot, N., and Duffau, H.	2010	Crossed aphasia elicited by intraoperative cortical and subcortical stimulation in awake patients	Vassal et al., 2010
30	Duffau, H., Leroy, M., and Gatignol, P.	2008	Cortico-subcortical organization of language networks in the right hemisphere: An electrostimulation study in left-handers	Duffau et al., 2008
31	Sanai, N., Zahman-Mizradeh, M.D., and Berger, M.S.	2008	Functional outcome after language mapping for glioma resection	Sanai et al., 2008

For the analysis of these articles, a two-phase procedure was conducted. In the first phase, the methodological characteristics of the 31 articles were systematically evaluated, specifying the characteristics of the samples, the histopathology of patients, the location of the lesion within the right hemisphere, the instruments used during intraoperative mapping, how the neuroanatomical data were acquired, and the neuropsychological assessment method used (Table 2). In the second phase, the information presented in the results sections of each article was synthesized (Tables 3, 4).

**Table 2:** Main methodological information of the reviewed articles.

**Cod**.	**Sample size (*n* =) (loc. RH)/left-handed (*n* =)**	**Main age years ±SD**	**Lesion**	**RH location**	**Techniques used**	**Neuropsychological functions monitored**
1	14/1	44.5 ± 13.2	LGG	Prefrontal areas (excluding frontal lesions invading the fronto-insular area and patients with premotor lesions)	DES, MRI	Spatial cognition, executive functions, and verbal semantic and phonological processing
2	22/no details	39.0 ± 1 2.19	LGG	Parietal lobe	MRI, DES	Cognitive flexibility
3	33/no details	48.0 ± 13.1	LGG	Frontal lobe (excluding primary motor area)	DES, MR	Visuospatial cognition, spatial working memory
4	22/2	42.8 ± 15.6	LGG, HGG	Frontal areas	DES, MRI	Basic emotions
5	15/no details	53.0 ± 9.78	LGG	Parietal, frontal, temporal, and insular regions	DES, 3D-MRI	Phonological and verbal semantic processing, spatial and social cognition, executive functions
6	111/13	39.8 ± 10.4	LGG	Supratentorial diffuse LGG close to or infiltrating at least in part the IFOF in the right hemisphere	DES, MRI,	Mentalizing, visual semantic cognition
7	256/24	38.7 ± 10.3	LGG	Frontal, temporal, frontal-temporal-insular, frontal-insular, parietal, temporal-insular, temporal-parietal, insular, frontal-parietal and temporo-occipital lobes	DES, MRI	Motor skills, phonological and verbal semantic processing, spatial cognition, mentalizing, speech and language, non-verbal semantic cognition
8	63/0	43 ± 14	LGG, HGG	Frontal, frontal-temporal, frontal-insular, frontal-insular-temporal, frontal-temporal-parietal, temporal-insular, temporal-insular-frontal	DES, MRI, DTI	Executive functions
9	117/14	39.0 ± 10	LGG	Frontal areas, frontotemporo-insular region, temporal areas	DES	Motor skills
10	16/0	35.53 ± 8.91	LGG	SMA, frontotemporo-insular area, frontal lobe, temporoinsular region, frontoinsular area, insular lobe	DES	Verbal semantic processing and motor skills
11	4/1	34.0 ± 8.18	LGG	Superior frontal gyrus	3DT1, DES, MRI	Motor function, working memory
12	20/no details	47.4 ± 10.3	LGG, HGG	Frontal, parietal, and temporal lobes	DES, MRI	Mentalizing
13	92/7	38.7 ± 10.5	LGG	Fronto-temporo-insular areas	DES, fMRI, DTI	Mentalizing, ToM
14	45/no details	37.8 ± 11.9	LGG, HGG	Frontal lobe	DES, 3DTI, MRI	Executive functions
15	14/1	44.0 ± 13.67	LGG, HGG	Inferior parietal lobe	DES, 3DT1	Somaotsensory functions, visuospatial cognition, motor skills, verbal semantic and phonological processing
16	49/7	–	LGG, HGG	Parietal and frontal lobes	DES, MRI	Motor skills
17	4/0	44.5 ± 5.4	LGG, cavernous angiomas	Frontal lobe and supplementary motor areas	DES, MRI	Verbal semantic and phonological processing
18	13/1	44.7 ± 12.8	LGG	Right ventral semantic network	MRI, DES	Verbal semantic processing
19	18/no details	46.6 ± 11.1	LGG, HGG	Frontal and parietal lobes	DES, MRI	Working memory, visuospatial cognition, processing speed, emotion, semantic processing
20	27/no details	39.3 ± 10.3	LGG	Frontal, temporal and fronto-temporo-insular areas	MRI, DTI, DES	Mentalizing
21	10/2	31.9 ± 9.5	LGG	Near the dPMC, SMA, head of the caudate nucleus, anterior cingulate cortex, anterior arm of the internal capsule	MRI, DES	Motor skills
22	20/no details	38.9 ± 11.53	LGG	Temporo-insular areas, frontal lobe, temporo-occipital lobe, insular lobe and fronto-temporo-occipital regions	MRI, DTI, DES	Visuospatial cognition, executive functions
23	93/no details	38.35 ± 10.31	LGG	Anteroposterior trajectories of the associative white matter bundles	MRI, DTI, DES	Mentalizing
24	5/3	36.0 ± 10.2	LGG	Pre-supplementary motor areas	DES	Motor skills and speech articulation
25	46/no details	46.95 ± 15.54	LGG, HGG, cavernoma	–	MRI, DTI, DES	Spatial neglect, reading, and writing skills
26	165/22	38.7 ± 10.4	LGG	Frontal, parietal, temporal, and occipital areas	DES	Motor skills, speech articulation, semantic processing
27	18/2	47.0	LGG, HGG, metastatic lesions, arteriovenus malformations, malignant meningioma	Frontal, parietal, temporal lobes and paracentral areas	DES	Sensorimotor processing, verbal semantic processing, reading skills, visuospatial cognition, facial emotion recognition
28	29/no details	58.2 ± 9.65	LGG, HGG, metastatic lesions	Primary motor area (M1)	fMRI, DTI, DES	Spatial neglect
29	3/0	35.0 ± 9.16	LGG	Fronto-temporo-insular areas and temporal lobe	fMRI, DES	Verbal semantic and phonological processing
30	9/9	34.0 ± 6.54	LGG	Fronto-mesial paracentral areas, middle frontal gyrus (posterior part), inferior frontal gyrus (posterior part) + rolandic operculum + insula, inferior parietal lobule and temporal lobe (excluding the superior temporal gyrus)	MRI, DES	Verbal semantic processing
31	5/no details	41.2	LGG, HGG	–	MRI, DES	Verbal semantic and phonological processing

DES, Direct electrical stimulation; dPMC, dorsal premotor cortex; DTI, Diffusion tensor imaging; fMRI, Functional magnetic resonance; HGG, High-grade glioma; LGG, Low-grade glioma; MRI, Magnetic resonance imaging; RH, Right hemisphere; SMA, Supplementary motor area; 3D-MRI, Three-dimensional magnetic resonance imaging.

**Table 3:** Distribution of the brain areas (cortical and subcortical levels) and the percentage of tasks systematically used in each phase (pre-, intra-, and post-operative) per cognitive functions.

**RH functions**	**Cortical anatomical distribution**	**Subcortical anatomical distribution**	**Intraoperative tasks**	**Pre- and post-op tasks**
Language and speech	DLPFC Frontal operculum STG Precentral gyrus Supramarginal gyrus vPMA MTG IFG Postcentral gyrus M1	FAT IFOF FST SLF II SLF III AF	$\begin{array}{l} \text{DO}80\text{ task }(n\;=\;10;\;90.9\%)\\ \text{PPTT }(n\;=\;7;\;63.63\%) \end{array}$ Counting tasks (*n* = 4; 36.36%) Naming tasks (*n* = 4; 36.36%) Reading and writing tasks (*n* = 2; 18.18%)	Fluency task (*n* = 4; 40%) Naming task (*n* = 3; 30%) DO80 task (*n* = 3; 30%) Reading and writing tasks (*n* = 2; 20%) The Boston Aphasia Examination (*n* = 1; 10%) Counting task (*n* = 1; 10%) SLTA (*n* = 1; 10%) MECP (*n* = 1; 10%) Token test (*n* = 1; 10%)
Spatial cognition	SFG MFG Supramarginal gyrus DLPFC IFG STG Angular gyri Postcentral gyrus	SLF II SLFIII IFOF AF FST	$\begin{array}{l} \text{Line bisection task }(n\;=\;10;\\ 100\%) \end{array}$ Cancellation tasks (*n* = 1; 10%) Milner Landmark test (*n* = 1; 10%)	Line bisection task (*n* = 5; 83.33%) *N*-back test (*n* = 2; 33.33%) Clock drawing (*n* = 2; 33.33%) Bell cancellation (*n* = 2; 33.33%) Letter cancellation task (*n* = 1; 16.67%) Raven matrices (*n* = 1, 16.67%) Figure copy (*n* = 1; 16.67%) VSOP (*n* = 1; 16.67%)
Social cognition	IFG DLPFC STG PMA SMA MFG SFG Prefrontal cortex Cingulum	IFOF SLF III AF FAT FST	RME (n = 9; 70.31%) Expression recognition test (*n* = 3; 23.44%) False belief (*n* = 1; 7.81%) Comic strips (*n* = 1; 7.81%) Japanese facial expression of basic emotion series by Matsumoto and Ekman (1989) (*n* = 1; 7.81%)	RME (*n* = 3; 60%) WAIS III PA (*n* = 1; 20%) The Expression Recognition Test for Adults by Komatsu et al. (2012) (*n* = 1; 20%)
Motor/sensory functions	Precentral gyrus vPMA M1 SMA S1	FST FAT AF SLF II SLF III White matter fibers of M1 and SMA	$\begin{array}{l} \text{Dual-task }(\text{DO80 task }+\text{ limb}\\ \text{movements})\;(n\;=\;6;\;75\%)\\ \text{Movement of upper and lower}\\ \text{limbs }(n\;=\;4;\;50\%) \end{array}$ Sensorimotor tasks (*n* = 3; 37.5%) Hand motor task (HMt) (*n* = 2; 25%) Simple test for evaluating hand function (*n* = 1; 12.5%)	Movements of the upper and lower limbs (*n* = 4, 80%) The Hand Strength Scale (*n* = 1; 20%)
Executive functions	DLPFC SFG	AF SLF III FAT	$\begin{array}{l} N-\text{back test }(n\;=\;5;\;71.42\%)\\ \text{Stroop test }(n\;=\;4;\;57.14\%) \end{array}$ TMT A/B (*n* = 2; 28.57%) Digit span test (*n* = 2; 28.57%) Short-term memory evaluation (*n* = 1; 14.28%) Adapted version of Stroop test (iST) (*n* = 1; 14.28)	*N*-back test (*n* = 3; 42.86%) Stroop test (*n* = 3; 42.86%) TMT A/B (*n* = 3; 42.86%) Rey-Osterrieth complex figure (*n* = 2; 28.57%) Cognitive estimations (*n* = 1; 14.28%) Digit symbol substitution (*n* = 1; 14.28%) WMS-R (*n* = 1; 14.28%)

Shaded Yellow color: Systematized intraoperative tasks in more than 50% of the cases. The *n* is the number of times the task has been used. MECP, Montreal Evaluation of Communication protocol; SLTA, standard language test of aphasia; VSOP, Visual and Object Space Perception battery; WAIS III PA, Picture arrangement task of the Weschler Adult Intelligence Scale. Third edition; WMS-R, Weschler Memory Scale-Revised Edition. Cortical and subcortical structures are organized in a descending way based on their frequency of appearance in the reviewed articles.

**Table 4:** Relevant results of the reviewed studies.

**Cod**.	**Pre-op neuropsychological assessment**	**Intraoperative tasks**	**Cortical positive sites**	**Subcortical positive sites**	**Post-op neuropsychological outcome and resection percentage**
1	Raven matrices; TMT A/B; cognitive estimations; Stroop test; digit symbol substitution test; digit span; o'clock test; figure copy; line bisection task; Rey-Osterrieth complex figure test (Executive functions deficits)	Stroop test; Short-term memory; working memory; Narrative language; SDMT; Milner landmark test; IAPS; Attentional matrices	Frontal lobe	–	• One week after resection, the greatest differences were found in the clock drawing test (100–77.7%) and cognitive estimation (100–72.7%). •*Resection percentage:* 95.53%
2	TMT; Stroop test (Cognitive flexibility deficits and non-motor neglect)	TMT; SCWDT	–	AF, SLF III	• Five out of 22 patients exhibited a decline in performance on the post-operative assessment of TMT compared to the pre-operative assessment. There was no observed improvement in TMT performance following glioma surgery. On the SCWDT, the performance of two patients worsened, while one patient showed improvement after glioma surgery (3–18 months after resection). •*Resection percentage:* No details
3	Spatial 2-back test; line bisection test (Impairment of visuospatial cognition and spatial working memory)	Line bisection task; Spatial 2-back test	MFG	White matter tracts of pMGF and pSFG involving the cingulum	• Patients with intraoperative line bisection test 3 months post-op had better performance than the group without it (5.6 vs. 33.3%). Regarding the SWM, the number of patients with deficits in this task decreased in both the group with and without intraoperative testing. However, the improvement was significant in patients who underwent testing during DES. •*Resection percentage:* removal extent better for VSC.
4	Expression recognition test for adults (Komatsu et al., 2012) (Basic emotion deficits)	Photographs of modified Japanese facial expressions of basic emotion series (Matsumoto and Ekman, 1989)	PMA, prefrontal cortex, MFG, SFG, IFG	–	• The score on the basic emotion test 1 week after surgery decreased in 9 out of 22 patients. However, optimal performance on the task was regained within 3 months after surgery. •*Resection percentage:* Total resection (100%) in 2 cases.
5	–	Number counting; DO80 oral picture naming task; PPTT; dual task; Line bisection task; RME	Insular lobe, frontal lobe, parietal lobe	–	• At 6 months post-surgery, only one patient worsened, experiencing a decline in performance in naming tasks and semantic tasks. •*Resection percentage:* 86.66%
6	Non-mentalizing and non-visual semantic cognition damage	RME; PPTT; manual version of line bisection task; naming task; motor tasks; somatosensory passive tasks	DLPFC, STG, IFG	IFOF, white matter fibers of the posterior parietal cortex	• – •*Resection percentage:* 92.8%
7	–	Simple and complex motor tasks; counting task; repetition test; DO80 task; PPTT; line bisection task; RME	Pre-central gyrus, post-central gyrus, PMC, SMA, VPMA, pre-SMA, DLPFC, MFG, SMG, SFG, MTG, IFL, IPL, IFG, AG	SLF II, SLF III, AF, IFOF, UF	• – •*Resection percentage*: No details
8	Token test, naming; semantic fluency; phonemic fluency; attentive matrices; TMT A/B; Stroop test; digit span test; Rey-Osterrieth complex figure test; Raven matrices (non-Stroop test deficits)	Stroop test; TMT A/B; counting task; naming test	IFG, posterior region of the IFG, pars opercularis, pars triangularis, MFG	SLF II, SLF III, FAT, FST	• In the control group, there was a decline in performance for TMT A/B, and there was a slower improvement in the Stroop test after surgery. In the iST group, improvements were observed over time (1 month follow-up). •*Resection percentage:* 100%
9	Non-motor disturbances	DO80 object naming; movement of upper and lower limb	Precentral gyrus, SFG, vPMA,	AF, FST, SLFII, SLF III, FAT	• Non-permanent motor impairments were observed after 3 months post-surgery. •*Resection percentage:* No details
10	DO80 task; PPTT (non-verbal and non-verbal semantic processing deficits)	Naming task + PPTT; naming task (DO80)	Posterior DLPFC, pars opercularis	–	• –•*Resection percentage:* No details
11	SLTA; WAIS-III; WMS-R; FAB; movement of upper and lower limb (Motor and phonological deficits)	Movement of upper and lower limb; digit span test; N-back test; line bisection task	DLPFC	FAT	• Six months after surgery, the average quotient of verbal intelligence and comprehension significantly improved for all WAIS-III tests. The mean scores of WMS-R for general memory and delayed recall also improved. No statistically significant differences were found in the mean scores of the FAB. •*Resection percentage:* Total resection (100%)
12	WAIS-III PA task (Impairments in high-level mentalizing)	WAIS-III PA task; The false belief task	–	FAT, FST, SLF III	• The accuracy of both high-level and low-level mentalizing in patients was lower compared to that of healthy volunteers prior to surgery and 1 week post-surgery. However, optimal performance on mentalizing tasks was regained within 3 months after surgery. •*Resection percentage:* 75.7%
13	RME (Mentalizing and ToM deficits)	Picture-naming; PPTT; manual version of line bisection test; RME	–	AF, IFOF	• – •*Resection percentage:* 59.11%
14	Raven matrices; alternative matrices; TM; verbal fluency; digit span; Stroop task (Non language and visual deficits)	Adapted version of the Stroop task (iST)	–	White matter tracts underlying the IFG, MFG, and the anterior insula	• Reduction of executive function deficits seven days post-op in the iST group. •*Resection percentage*: 85.2%
15	The bell test; line bisection task (non-visuospatial cognition deficits)	DO80 naming test; dual-task; modified version of the picture naming task; manual version of line bisection task; PPTT	Posterior supramarginal gyrus, MTG	SLF II, SLF III, IFOF, AF	• Immediately after surgery (5 days later), two out of 14 patients had spatial neglect, one left hemianopia, and three left superior quadrantanopia. Three months after surgery, these impairments disappeared. •*Resection percentage:* No details
16	Non-hand motor, praxis, somatosensory or visual deficits	Token test; picture naming; verbal fluency; digit span; Rey-Osterrieth complex figure; Raven Matrices; Stroop test, TMT	Ventrolateral premotor cortex, M1	White matter tracts underlying M1, SMA, and ventrolateral premotor cortex	• The group that used the intraoperative Hand Motor Task had a significantly lower incidence of ideomotor apraxia 1 week after surgery. •*Resection percentage:* 93.3%
17	Speech articulation deficits (speech arrest)	Counting task; PPTT	STG, inferior frontal cortex, supramarginal gyrus	SLF III	• Prediction of SAN through pre-surgical ICA fMRI yielded promising results, as evidenced by the moderate degree of correlation between the connectivity of SAN seed regions and the GOF of the speech articulation component obtained. •*Resection percentage:* No details
18	PPTT; naming task (none of the patients experienced verbal or non-verbal semantic disturbances)	PPTT; DO80 task	DLPFC, STG, pars opercularis, pars triangularis	IFOF	• Five days after surgery, all patients scored above the cutoff on the PPTT and DO80 tasks. •*Resection percentage:* No details
19	Letter cancellation test; verbal fluency test; spatial 2-back test; RME; WAIS III PA; line bisection test (Visuospatial cognition, working memory, and ToM impairments)	Dual-task; 2 N-back test; Stroop test; expression recognition test; ToM test; line bisection test	SFG	SLF II, SLF III	• In the normal group, several cognitive abilities (SP, WM, and ToM) recovered at 3 months, except VSC, which persisted in 33.3% of patients. In the deficit group at 3 months, 80% showed improvement. However, 6 out of 18 patients continued to have visuospatial deficits even 1 year after surgery. •*Resection percentage:* eight gross total resections, four subtotal resections, six partial removals.
20	RME (Mentalizing deficits)	RME; PPTT; DO80 test; manual version of line bisection task	DLPFC, IFG	IFOF	• There was a significant decline in mentalizing performance immediately after surgery, but this decline was temporary, as the performances prior to surgery did not show a statistically significant difference compared to those 3 months after surgery. •*Resection percentage:* 93.9%
21	Movement of upper and lower limb; naming task (none of the patients had motor or language disturbances)	DO80 object naming task; movement of upper and lower limb	M1	White matter tracts from the dPMC and SMA	• Slight left or right upper limb paresis and speech disorder (verbal fluency) that disappeared 3 months after surgery. •*Resection percentage:* No details
22	Stroop task; “Letters” and “Cube analysis” subtest of VSOP (attentional and visuospatial deficits)	Cancellation tasks (manual version of line bisection task; line cancellation; letter cancellation; bell cancellation, and apple cancellation)	–	SLF II	• The patients worsened in lateralized target detection, they made fewer omissions in visuospatial tasks, and no significant results were found in the line bisection task 2–4 days after surgery. •*Resection percentage:* 94.4%
23	Mentalizing impairments	RME; comic strap task	SMA, IFG, MFG	AF, SLF	• – •*Resection percentage:* 94.29%
24	Verbal fluency task; DO80 task; speech and movement of extremities (speech and motor initiation disturbances)	Dual-task combining DO80 naming test and motor actions	–	FAT, FST	• Some patients experience ‘initiation disturbances' (in speech, movements, or both) on the fifth day after resection. Three months after surgery, these deficits disappeared. •*Resection percentage:* Supratotal resection (>100%)
25	Bell cancellation test; clock drawing; line bisection task; text reading, writing; a representational task based on a map of France (Spatial neglect)	Line bisection task	STG, MTG, IFG supramarginal gyrus, angular gyri, postcentral gyrus	AF, IFOF, SLF II	• 6 out of 41 (14.6%) experienced post-operative spatial neglect observed between 2 and 8 weeks after surgery. •*Resection percentage:* Gross total resection.
26	Non-speech disturbances	Counting; DO80 picture naming task; dual task	Precentral gyrus, VPMA, pars opercularis, pars triangularis, STG, MFG, precentral and postcentral gyri, DLPFC	–	• – •*Resection percentage:* No details
27	Written and oral understanding; naming; language fluency and object handling; visual field (no spatial neglect)	Manual version of line bisection task; object naming task; sensorimotor task and facial emotion task; naming and reading tasks.	T1, T2, supramarginal gyrus, frontal, parietal, angular gyrus	–	• Post-operative impairments were identified. One out of 18 experienced minor post-operative prosopagnosia, and three patients had post-operative spatial neglect. Among these patients, the neglect persisted during the 3-month follow-up neuropsychological evaluation for two individuals. •*Resection percentage:* No details
28	Hand strength scale (non-motor hemineglect)	A simple test for evaluating hand function	M1, S1	–	• Two out of 29 developed motor hemineglect. •*Resection percentage:* No details
29	Fluency task; the Boston Diagnosis Aphasia Examination; DO80 picture naming test; MECP (anomia and verbal semantic paraphasia)	DO80 picture-naming task	M1, PMA, STG, IFG	–	• In two out of three patients, the postsurgical course was uneventful, with no neurological worsening. The language assessment 4 days after surgery show no abnormalities. One patient's transient language worsening (anomia) occurred 24 h after surgery. •*Resection percentage:* No details
30	The Boston Diagnostic Aphasia Examination (Non-Speech disturbances or semantic and phonological impairments)	Sensorimotor tasks; DO80 task	PMC, DLPFC, IFG, pars ocpercularis, supramarginal gyrus, STG, MTG	SLF, IFOF	• Nine patients had transient language disorders (mutism, phonetic paraphasia, anarthria, semantic disturbances). These deficits disappeared between 7 days and 3 months after surgery.•*Resection percentage:* No details
31	Counting; naming objects; reading single words; repeating complex sentences; writing words and sentences on a paper (speech arrest, anomia, and alexia)	Counting numbers; naming objects, and reading single words	Frontal, parietal lobes	–	• All those patients who developed language deficits between 1 and 6 weeks after surgery return to the baseline function or, better 3 months after resection. Only two out of five developed motor hemineglect. •*Resection percentage:* 59.6%

AF, Arcuate fasciculus; AG, Angular gyri; DLPFC, Dorsolateral prefrontal cortex; DO80, Test de Dénomination Orale d'images; FAB, Frontal assessment battery; FAT, Frontal aslant tract; FST, Fronto-striatal tract; IAPS, International Affective Picture System; GOF, Goodness of fit; ICA, Independent component analysis; IFG, Inferior frontal gyrus; IFL, Inferior frontal lobe; IFOF, Inferior fronto-occipital fasciculus; IPL, Inferior parietal lobe; M1, Primary motor area; MECP, Montreal Evaluation of Communication protocol; MFG, Middle frontal gyrus; MTG, Medial temporal gyrus; PMA, Premotor area; PMC, Primary motor cortex; pMFG, posterior middle frontal gyrus; PPTT, Pyramids and Palm Tree Test; pSMG, posterior superior frontal gyrus; pSTG, Posterior superior temporal gyrus; RME, Reading the Mind in the Eyes; S1, Primary somatosensory area; SAN, Speak articulation network; SCWT, Stroop Color-Word Test; SDMT, Symbol Digit Modalities Test; SFG, Superior frontal gyrus; SLF, Superior longitudinal fasciculus; SLTA, standard language test of aphasia; SP, Speed processing; STG, Superior-temporal gyrus; SWM, Spatial working memory; T1/T2, Parts of the temporal lobe; TMT, Trail Making Test; ToM, Theory of Mind; TPJ, Temporo-parietal junction; UF, Uncinate fasciculus; vPMA, ventral premotor area; VSC, Visuospatial cognition; VSOP, Visual Object and Space Perception Battery; WAIS-III, Weachler Adult Intelligence Scale. Third version; WAIS III PA, Picture arrangement task of the Weschler Adult Intelligence Scale; WM, Working memory; WMS-R, Weschler Memory Scale-Revised Edition.

### 2.2 Inclusion and exclusion criteria

The inclusion and exclusion criteria were defined according to the PICO (patient population, intervention, control, outcome) framework criteria. All articles were included that reported on adult patients with gliomas of the right hemisphere (WHO grade I–IV: patient population) who underwent awake craniotomies with DES (intervention) and who produced specific intraoperative cognitive errors (outcome) while stimulating or resecting in a specific reported brain location (outcome). As long as DES was used, studies using additional imaging techniques (e.g., intraoperative Magnetic Resonance Imaging, iMRI; Diffusion Tensor Imaging, DTI; functional Magnetic Resonance Imaging, fMRI) were also included.

Articles were excluded if brain locations or cognitive errors were not reported, not further specified, or not clear. Articles were also excluded if they did not report (original) patient data or intraoperative neuropsychological information on glioma patients or if the article was a book chapter, comment, case report, editorial, or was written in a language other than English. The PRISMA flowchart is shown in Figure 1.

### 2.3 Data analysis

The different data analyses are explained below.

#### 2.3.1 First analysis: cortical and subcortical distribution of brain functions assessed

The cognitive functions and a percentage of how often these cognitive processes occurred in a specific brain location (cortically and subcortically) were calculated based on the total number of articles that reviewed each cognitive function (e.g., total of speech articulation articles: *n* = 7, speech arrest in location x: *n* = 3 =42.85%).

Locations, including the gyrus, cortex, and lobe, were seen as cortical. At the subcortical level, a distinction was made between general subcortical locations (e.g., white matter below primary motor area, M1 and supplementary motor area, SMA) and subcortical tracts (e.g., inferior fronto-occipital fasciculus, IFOF; superior longitudinal fasciculus SLF).

#### 2.3.2 Second analysis: cognitive functions and intraoperative tasks

Individually, for each cognitive function, a percentage of how often an intraoperative task was used was calculated based on the total number of articles that reviewed each cognitive process (e.g., total spatial cognition articles: *n* = 9, cancellation task performed in *n* = 1 articles = 11.11%). The level of evidence for each task was also compiled. A high level of evidence was considered for a task when its frequency and percentage of occurrence were equal to or >50%.

#### 2.3.3 Third analysis: pre- and post-operative outcomes

For each cognitive function, a percentage was calculated to indicate how frequently a task was used pre- and post-operatively. This calculation was based on the total number of articles that assessed each cognitive function in the pre- and post-op phases.

### 2.4 Participant characteristics

In Table 2, the main methodological information is synthesized. In 25 articles (1, 2, 3, 5, 6, 7, 8, 9, 12, 13, 14, 15, 17, 19, 20, 22, 23, 24, 25, 26, 27, 28, 29, 30, and 31), the data were collected in order to study the effects of the transitory disconnection of different brain areas caused by DES and its effects on the cognitive functions (semantic and phonological processing, speech articulation, visuospatial cognition, working memory, and cognitive flexibility) and in six studies (4, 6, 10, 11, 16, and 19) DES caused transient disturbances in socio-cognitive processing [mentalizing, theory of mind, (ToM), and basic emotion deficits].

The sample size in Table 2 indicates the number of subjects who had a lesion within the right hemisphere. The total number of participants included in this review is 1,358; although most studies had a sample of fewer than 50 participants, five studies include less than six patients (11, 17, 24, 25, and 31), and only six publications evaluated 92 to 245 cases (6, 7, 9, 13, 23, and 26). The mean age of the participants ranges from 34.0 to 58.2 (range: 18–63 years). Left-handed participants were also considered in this study. Only five out of 31 articles selected for the present systematic review showed a left-handed sample size >3 (6, 9, 16, 26, and 30). Articles 10, 17, and 29 only include right-handed patients in their studies, and 11 articles (2, 3, 5, 12, 19, 20,22, 23, 25, 28, and 31) did not report the handedness of the subjects.

The location of the lesion within the right hemisphere was specified in 12 studies (37.03%) (3, 6, 10, 11, 14, 15, 16, 17, 18, 21, 23, 24, 28, and 30). Two articles (23 and 29) did not report the location, and 13 (1, 2, 4, 5, 9, 12, 13, 19, 20, 22, 26, 27, and 29) only showed the cerebral lobe and lobule regions where the glioma was located (frontal, parietal, temporal, insular, and occipital lobes).

The main lesion located in the right hemisphere was the diffuse low-grade glioma (LGG; WHO grade II). In addition to LGG, high-grade gliomas (HGG; WHO III and IV) were found in 33.33% (*n* = 11), and other lesions such as cavernous angiomas (17), cavernomas (25), metastatic lesions (27, 28), arteriovenous malformations (27), and meningiomas (27, 28) were described (*n* = 4; 14.81%).

Finally, all the studies used DES intraoperatively. Neuroimaging techniques such as MRI, fMRI, or 3D-MRI were used to analyze both the location and size of the lesion prior to surgery, as well as examine the volume of tissue resected after surgery. No MRI imaging techniques were conducted in four studies (9, 10, 26, and 27). Diffusion tensor imaging techniques were combined with MRI studies in ten articles (7, 8, 11, 13, 14, 17, 22, 24, 25, and 28) to track the main bundles of white matter tracts pre-operatively.

## 3 Results

**Figure 2:**
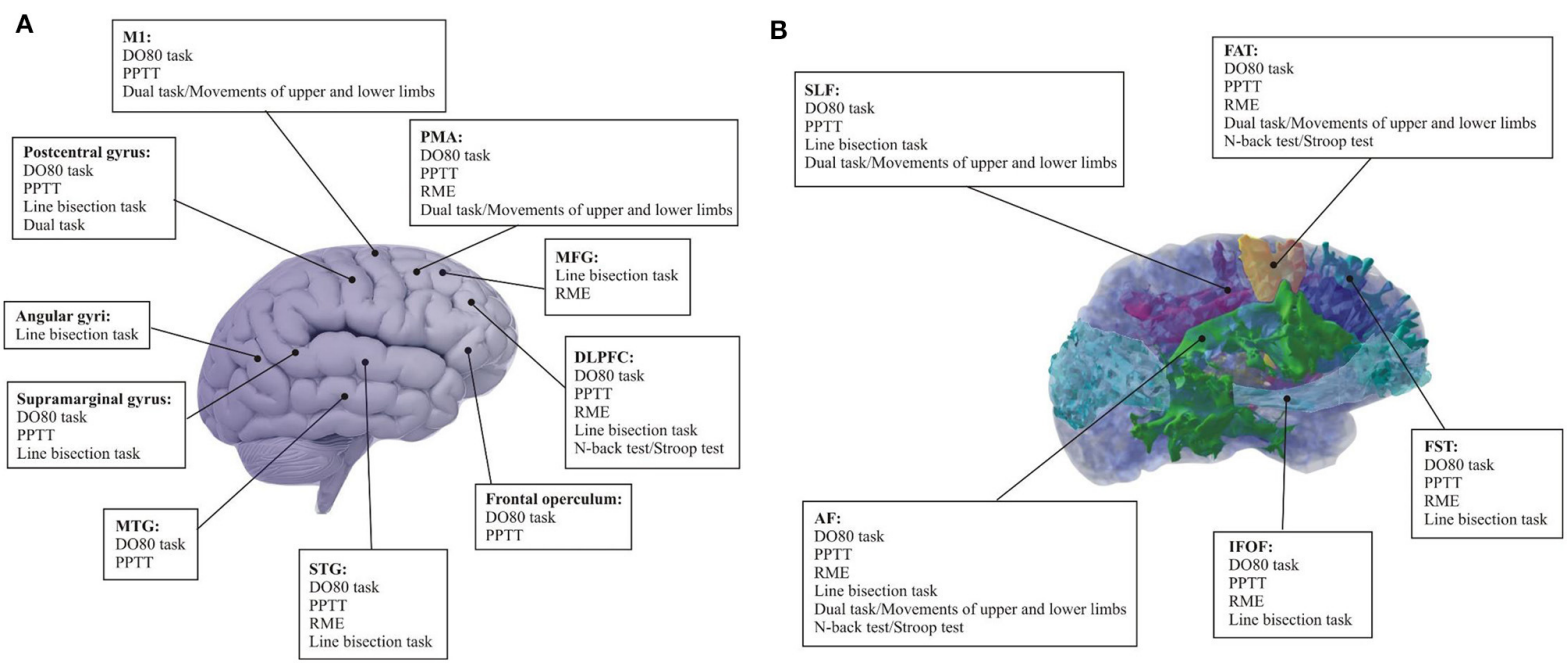
Summary of the neuropsychologically validated tasks frequently used and neural networks mediating cognitive and social function provided by cortico-subcortical electrical mapping. It shows cortical **(A)** and subcortical **(B)** visual representation. Note that only neuropsychological tasks with a high level of evidence (>50%) are indicated. D080 task (90.9%); PPTT (63.63%); Line bisection task (100%); RME (70.31%); Dual-task (75%); Movements of upper and lower limbs (50%); *N*-back test (71.42%); Stroop test (50%). AF, Arcuate fasciculus; DLPFC, Dorsolateral prefrontal cortex; DO80 task, Dénomination orale d'images test; FAT, Frontal aslant tract; FST, Fronto-striatal tract; IFOF, Inferior fronto-occipital fasciculus; MFG, Middle frontal gyrus; MTG, Middle temporal gyrus; M1, Primary motor cortex; PMA, Premotor area; PPTT, Pyramids and Palm Tree Test; RME, Reading the Mind in the Eyes; SLF, Superior longitudinal fasciculus; STG, Superior temporal Gyrus.

This review article presents an in-depth analysis of the 31 articles included in Table 1. In the search for which cognitive and emotional processes have been related to positive cortical-subcortical sites when right brain surgery has been performed, as well as which neuropsychological tasks are more sensitive to assess high-order cognitive processing during surgery (Figure 2), we performed a systematic analysis of 1. neuropsychological functions assessed during pre-, intra- and post- right awake surgery; 2. type of tasks employed during right awake surgery; and 3. Pre- and post-surgical neuropsychological outcomes (Table 4). Figure 1 presents the highlights of this literature review.

### 3.1 First analysis: cortical and subcortical distribution of brain functions assessed

As shown in Table 3, the 31 articles selected for this systematic review comprehensively explore different neurocognitive functions that were assessed during DES in right awake surgery patients. The included studies predominantly assessed essential cognitive domains, including language, spatial cognition, social cognition, motor/sensory functions, and executive functions. The relationship between these cognitive functions and cortical and subcortical brain regions was analyzed.

#### 3.1.1 Language and speech

Language and speech functions were assessed in 12 of the 31 articles selected for the present systematic review (41.34%).

Speech arrest disturbances were described cortically in seven articles (53.84%): Ventral premotor area (vPMA) (*n* = 4; 57.14%), the precentral gyrus (*n* = 4; 57.14%), frontal operculum (pars opercularis and triangularis; *n* = 3; 42.85%), supramarginal gyrus (*n* = 3; 42.85%), medial temporal gyrus (MTG) (*n* = 2; 42.85%), inferior temporal gyrus (IFG) (*n* = 2; 42.85%), dorsolateral prefrontal cortex (DLPFC) (*n* = 2; 14.28%), superior temporal gyrus (STG) (*n* = 1; 14.28%), and primary motor area (M1) (*n* = 1; 14.28%). Five (7, 8, 9, 24, 30) of the seven articles showed the results of DES at the subcortical level: superior longitudinal fasciculus II (SLF II) (*n* = 4; 80%), superior longitudinal fasciculus (SLF III) (*n* = 3; 60%), frontal aslant tract (FAT) (*n* = 1; 33.33%), fronto-striatal tract (FST) (*n* = 1; 100%), arcuate fasciculus (AF) (*n* = 1; 33.33%), and IFOF (*n* = 1; 33.33%).

Verbal and non-verbal semantic cognition were also analyzed (*n* = 7; 58.33%). The cortical regions involved in semantic cognition were as follows: DLPFC (*n* = 5; 83.33%), STG (*n* = 3; 50%), frontal operculum (*n* = 2; 33.33%), MTG (*n* = 1; 16.67%), middle frontal gyrus (MFG) (*n* = 1; 16.67%), and IFG (*n* = 1; 16.67%). The subcortical areas related to semantic cognition were only described in four of the six articles (6, 9, 14, 26). The main white matter tract involved in this language component was the SLF III (*n* = 3; 75%) and IFOF (*n* = 2; 50%). FAT (*n* = 1; 25%) and was also found. Article 6 only reported that semantic cognition deficits appeared when DES was applied to the white matter tracts of the posterior parietal lobe. It should be noted that one article did not specify the positive language-related sites (1), and another one (5) showed that language disturbances were observed after the electrical stimulation of the insular and frontal lobes.

#### 3.1.2 Spatial cognition

Spatial cognition was analyzed in 10 articles of a total of 31 (34.48%). Two studies (13 and 20) assessed this neurocognitive function, but as part of the awake brain surgery routine. Eight out of the ten articles provided results about the intraoperative findings at the cortical level: MFG (*n* = 2; 20%), superior frontal gyrus (SFG) (*n* = 2; 20%), supramarginal gyrus (*n* = 2; 20%), the postcentral gyrus (*n* = 2; 20%), STG (*n* = 2; 20%), DLPFC (*n* = 1; 10%), IFG (*n* = 1; 10%), MTG (*n* = 1; 10%), SFG (*n* = 1; 10%), inferior parietal lobe (IPL) (*n* = 1; 10%), and the angular gyri (*n* = 1; 10%). The subcortical positive sites were located mainly in SLF II and III (*n* = 10; 100%). Other significant white matter tracts involved in this cognitive process were IFOF (*n* = 2; 20%), FAT (*n* = 1; 10%), and AF (*n* = 1; 10%).

#### 3.1.3 Social cognition

Eight out of 31 articles selected (27.59%) showed mentalizing and emotional processing deficits during DES over the following cortical sites: IFG (*n* = 6; 100%), premotor area (PMA) (*n* = 2; 33.33%); DLPFC (*n* = 2; 33.33%), STG (*n* = 2; 33.33%), SFG (*n* = 2; 33.33%), MFG (*n* = 1; 16.67%); SMA (*n* = 1; 16.67%); cingulum (*n* = 1; 16.67%), and prefrontal cortex (*n* = 1; 16.67%). These percentages have been calculated considering the number of studies in which the cortical areas were mentioned (*n* = 6). The subcortical mapping was described in five articles (6, 12, 13, 18, and 21): IFOF (*n* = 3; 60%); SLF III (*n* = 2; 40%); AF (*n* = 2; 40%); FAT (*n* = 1; 20%), and FST (*n* = 1; 20%).

#### 3.1.4 Motor/sensory functions

Motor/sensory functions were evaluated in eight articles. Nevertheless, only five (9, 17, 21, 24, and 28) out of 31 reported results about intraoperative brain mapping (*n* = 5; 17.24%). The other three articles (11, 15, 19) carried out the assessment of these brain functions to avoid potential motor interferences in the cognitive processes they aimed to evaluate. Three studies (9, 21, 28) described the cortical brain mapping: M1 (*n* = 2; 66,67%), vPMA (*n* = 1; 33.33%), premotor cortex (PMC) (*n* = 1; 33.33%), SMA (*n* = 1; 33.33%), primary somatosensory area (S1) (*n* = 1; 33.33%), and precentral gyrus (*n* = 1; 33.33%); and all the articles described the subcortical brain mapping: FST (*n* = 2; 50%), FAT (*n* = 2; 50%), SLF (*n* = 2; 50%), AF (*n* = 1; 25%). One out of 4 (17) studies revised for the motor/sensory functions showed that motor skills were observed over the sensorimotor cortex during DES. No description of critical cortical or subcortical regions involved in this cognitive function was analyzed.

#### 3.1.5 Executive functions

Inhibition and working memory (executive functions) were found in 6 out of 31 articles (1, 2, 3, 8, 11, and 9) selected for the systematic review (20.69%). The cortical positive sites were: DLPFC (*n* = 1; 16.67%%) and SFG (*n* = 1; 16.67%); IFG (*n* = 1; 16.67%); and MFG (*n* = 1; 16.67%). At the subcortical level, FAT (*n* = 2; 33.33%) and SLF II and III (*n* = 2; 33.33%) were described. It should be noted that one article (1) suggested a non-specific brain mapping during DES.

### 3.2 Second analysis: cognitive functions and intraoperative tasks

The intraoperative findings at the cortical and subcortical levels for each of the cognitive functions assessed were made possible using different neuropsychological tasks (Tables 3, 4).

#### 3.2.1 Language and speech

A strong level of evidence showed that the main tasks used to assess semantic cognition and speech articulation intraoperatively were the Dénomination orale d'images test (DO80) (*n* = 10; 90.9%) and Pyramids and Palm Tree Test (PPTT) (*n* = 7; 63.63%). Other tasks were used. Nonetheless, their level of evidence was under 50%: naming task (*n* = 4; 36.36%), counting task (*n* = 2; 1.82%), and reading and writing tasks (*n* = 1; 0.9%).

#### 3.2.2 Spatial cognition

The line bisection test was the gold standard task used to evaluate spatial cognition (*n* = 10; 100%). A lesser level of evidence was found using cancellation tasks (*n* = 1; 10%) and Milner landmark test (*n* = 1; 10%).

#### 3.2.3 Social cognition

Mentalizing and emotional processing were mainly assessed by Reading the Mind in the Eyes (RME) (*n* = 9). This test showed the strongest level of evidence (70.31%). The expression recognition test (*n* = 3; 23.44%), the false belief (*n* = 1; 7.81%), comic strips (*n* = 1; 7.81%), and the Japanese facial expression of basic emotions series by Matsumoto and Ekman (1989) (*n* = 1; 7.81%) were also described in the reviewed articles. Notice that the latter task was used in one article (4) rather than RME. The authors argued that cultural biases influenced the interpretation of Western emotional gestures by the Asian participants in the study.

#### 3.2.4 Motor/sensory functions

The dual task (naming task + limb movements) showed a high level of evidence (75%) due to this task being performed in all articles that evaluated cognitive processes. Additionally, the movements of the upper and lower limbs (*n* = 4; 50%) showed a good level of evidence. A low level of evidence was found in sensorimotor tasks (*n* = 3; 37.5%). Finally, one article (28) provided the use of a simple test to evaluate hand function (*n* = 1; 12.5%), and another one (16) carried out intraoperatively a Hand Movement Task (HMt) (*n* = 1; 12.5%)

#### 3.2.5 Executive functions

The N-back test (*n* = 5; 71.42%) and Stroop test (*n* = 4; 57.14) showed a good level of evidence. In a low level of evidence, the digit span (*n* = 2; 28.57%), Trail Making Test A/B (TMT A/B) (*n* = 2; 28.57%), short-term memory test (*n* = 1; 14.28%), and adapted version of the Stroop test (iST) (*n* = 1; 14.28) were employed.

### 3.3 Third analysis: pre- and post-operative outcomes

In this section, the post-operative results are examined in relation to the baseline of each pre-operative case.

#### 3.3.1 Language and speech

Fourteen of the 31 selected articles for the systematic review included an assessment of semantic cognition and speech/language functions (*n* = 14; 48.27%) pre-operatively. Of these 14 articles, 11 described the pre-operative tasks used, and these included: fluency task (*n* = 4; 28.57%); naming task (*n* = 4; 28.57%); DO80 task (*n* = 3; 21.43%); reading and writing tasks (*n* = 2; 14.28%); the Boston Diagnostic Aphasia Examination (BDAE) (*n* = 1; 7.14%); counting task (*n* = 1; 7.14%); standard language test of aphasia (SLTA) (*n* = 1; 7.14%), and Montreal Evaluation of Communication protocol (MECP) (*n* = 1; 7.14%). Two articles (six and 13) reported semantic cognition and speech articulation disturbance (speech arrest) before surgery.

One article (7) did not describe the pre- and post-surgical examination. Similarly, one article (19) did not describe the pre-surgical examination. However, language post-op results were shared in article 19, with one patient showing a decline in naming and semantic tasks post-operatively. Articles 21 and 23 also described post-op decline in their sample. In article 21, there was a slight decline in verbal fluency tasks during the post-op assessment. In article 23, some patients experienced speech initiation disturbances post-op comparable to their original presentation during the days prior to surgery. Despite the initial worsening, all patients regained their linguistic skills at long-term follow-up (3 and 6 months post-op).

Transient language disorders such as mutism, phonetic paraphasia, anarthria, and semantic disturbances were observed in one article (30). While all patients who were normal pre-surgical developed speech or semantic language disturbance 24 hours after resection. These language disturbances disappeared by 7 days post-op. Three out of 13 articles (11, 18, 31) showed improvements in language tasks in comparison with the pre-op assessment between 1 week and 6 months post-op. Two studies (17 and 29) that reported speech arrest, anomia, and verbal semantic paraphasia pre-operatively experienced an improvement in language tasks immediately after surgery. Articles 6, 10, and 26 did not describe postsurgical outcomes, and article 27 did not examine the results of post-op language testing.

In addition to this information, it is important to note that five of the 13 (5, 6, 11, 24, and 31) articles that analyzed language pre and post-operatively reported results about the percentage of the brain tissue resected during the surgical procedure. In articles 11 and 24, a supratotal resection (> 100%) was performed. A gross resection was carried out in articles 5 and 6 (86.6 and 92.8%, respectively). In article 31, only 59.6% of the tumor was removed.

#### 3.3.2 Spatial cognition

Seven out of 31 articles performed spatial cognitive testing pre-operatively (25.93%). Three articles (3, 19, and 22) showed visuospatial and attentional deficits in the pre-operative period; two studies (14 and 16) reported non-spatial cognition impairments prior to surgery, and one (1) did not describe the results of the pre-op assessment in spatial cognition.

Among studies that reported on spatial cognitive testing, the most used task was the line bisection task (*n* = 5; 71.43%). To a lesser degree, clock drawing (*n* = 2; 28.57%) and bell cancellation (*n* = 2; 28.57%) were used. Only one article described the use of the letter cancellation task (*n* = 1; 14.28%), Raven Matrices (*n* = 1; 14.28%), figure copy (*n* = 1; 14.28%), and Visual Object and Space Perception Battery (VSOP) (*n* = 1; 14.28%).

The post-operative evaluation results were systematically described in all studies. Articles 3 and 19 described significant improvement pre- to post-operatively in visuospatial cognition tasks (Table 4). Improvement in line bisection was noted 3 months after surgery. Similar results were obtained in article 22, with improvement in spatial cognition tests reported.

Article 1 did not report any information about pre-operative neuropsychological outcomes, but the post-operative results were described. One week after the resection, there was a slight decline in performance on the clock drawing task (100–77.7%). This decline was also observed in articles 15 and 27. However, these disturbances were transient and resolved 3 months after the resection.

The extent of resection was described in five out of six articles that assessed spatial cognition pre- and post-operatively. The percentage of tissue resection was more than 90% in articles 1 (95.53%) and 22 (94.4%). Articles 19 and 25 only reported the type of resection (gross, subtotal, or partial resection).

#### 3.3.3 Social cognition

Social cognition was analyzed pre-operatively in seven of the 31 (*n* = 7; 25.92%) articles selected. Articles 12, 13, 20, and 23 showed pre-operative impairments in mentalizing, and one article (4) found basic emotion deficits prior to surgery. Only one study (6) did not report mentalizing disturbances. Five out of the 31 articles that assessed social cognition detailed the measures used. RME was the gold standard test used to evaluate this cognitive process (*n* = 3; 60%). Other tasks described included picture arrangement tasks from the Weschler Adult Intelligence Scale (WAIS-III PA) (*n* = 1; 20%) and the Expression Recognition Test for Adults by Komatsu et al. (2012) (*n* = 1; 20%).

The post-operative outcome was described in three of the articles reviewed. Four studies (11, 13, 19, and 23) did not report information about the neuropsychological follow-up. In articles 12 and 20, similar impairments in mentalizing pre- and 24 h to 1 week post-operatively were noted. Article 4 reported similar results. Nonetheless, all the disturbances exhibited in articles 12, 20, and 4 were transient, with improvements seen by 3 months post-op.

Regarding the extent of resection, one article (4) described a complete resection (100%) in two patients, and two articles (20 and 22) reported the removal of more than 90% of the tumor (93.3 and 94.29%, respectively). Article 12 reported 75.7% of the tumor resected, and article 13 noted 59.11% extent of resection.

#### 3.3.4 Motor/sensory functions

Motor/sensory functions were assessed pre-operatively in 18.52% (*n* = 6) of the articles reviewed in the present study. Two articles (11 and 24) reported motor impairment pre-operatively, while four articles (9, 16, 21, and 28) did not report any motor disturbances prior to surgery. Four out of six articles described the tasks used, which included the movement of the upper and lower limbs (*n* = 4; 80%) and the Hand Strength Scale (*n* = 1; 20%), and only one article (16) did not report the tasks used pre-operatively.

The post-operative outcome was described in all articles except article 11. All studies reported transient motor disturbances, including paresis; one article (16) described a significantly lower incidence of ideomotor apraxia 1 week post-op. Even in studies (9, 21, and 28) in which the pre-operative assessment did not describe motor impairments, temporary deficits were noted immediately after surgery. Nevertheless, these disturbances disappeared between 3 and 6 months post-op.

Only three studies (11, 16, and 24) reported the extent of resection (>93%) (see Language section and Table 4 for more information about articles 11 and 24).

#### 3.3.5 Executive functions

Executive functions, such as inhibition and working memory, were evaluated prior to surgery in 27.59% (*n* = 9) of the articles reviewed. One out of four studies (4) described cognitive flexibility deficits. One other article (1) reported more general executive function impairments, and article 14 described the absence of language and visual deficits. Articles 11 and 22 did not report executive function results pre-operatively.

The tasks used during the pre-operative phase were described in all the studies. The most used task was the N-back test, administered in four out of nine articles (50%). The Stroop test and the TMT were employed in 37.5 % (*n* = 3) of the articles. Digit symbol substitution (*n* = 2; 25%), digit span test (*n* = 2; 25%), Rey-Osterrieth complex figure (*n* = 2; 25%), cognitive estimations (*n* = 1; 12.5%), and the Weschler Memory Scale-Revised Edition (WMS-R) (*n* = 1; 12.5%) were used, but to a lesser degree.

Post-operative neuropsychological assessment was carried out in four articles. Only one study (22) did not report results on executive functions. One study (11) described an improvement in the mean scores of the WMS-R during the post-op phase (6 months after surgery), and another article (14) reported a reduction of executive function deficits 7 days post-op. Three studies (1, 2, 8) showed a decline in executive functions after surgery. One article (1) reported a worsening in cognitive estimations. In an article (2), 22.72% of patients exhibited a decrement in TMT. Similarly, 9.09% of patients showed worse performance on the Stroop test immediately after surgery. However, recovery was seen at 3 and 18 months post-op. Article 8 reported a worsening in Stroop performance with improvement by 1 month post-surgery.

Three articles (11, 14, and 22) showed resection data (>85%) (see Table 4 for more information).

## 4 Discussion

This study examines the neuropsychological assessment tools used to map right hemisphere functions during awake brain surgery over the past 15 years. It emphasizes the need for carefully chosen tasks for intraoperative assessment of right hemisphere functions and systematic use of pre- and post-operative assessments to enhance the understanding of cognitive and emotional processes related to right cortical-subcortical structures. The study contributes to a deeper appreciation of the functional anatomy of the right hemisphere for neurosurgeons and neuropsychologists participating in awake brain surgeries. What follows is a discussion of the most common functions evaluated and tasks used during right hemisphere resections.

### 4.1 Language and speech

Language and speech are by far the most tested cognitive domains in awake brain surgery. Almost 50% of the studies included in this review describe a test or test paradigm for the assessment of language and speech functions within the right hemisphere. Simple tasks like counting and more complex assessments such as semantic associations or translation skills are employed to monitor speech processing and language functions. Standardized neuropsychological tests, including DO80 (Deloche and Hannequin, 1997), are commonly used. The DO80 task, especially, exhibits strong evidence (81.81%) at the intraoperative level, effectively assessing a range of language disorders. For intraoperative assessment of verbal and non-verbal semantic cognition, PPTT is frequently employed (54.54%). In the study conducted by Herbet et al. (2018), PPTT was used in addition to the DO80 task. They wanted to analyze semantic paraphasia because the main aim of their study was to investigate the contribution of prefrontal structures to semantic processing but not to other language-related processes. Employing only the DO80 task was not enough to describe the semantic processing. Consequently, a combination of DO80 and PPTT is proposed as the gold neuropsychological standard to assess key language processes.

While language processing in the left hemisphere is well-established, the role of the right hemisphere remains debated. Evidence suggests the right hemisphere is involved in core language functions, challenging traditional views. The revisited model of language processing, proposed by Herbet and Duffau (2020), identifies a dual route: the dorsal phonological stream and the ventral semantic route. The dorsal stream comprises deep and superficial components. The deep component corresponds to the connections between the posterior temporal structures (MTG and ITG) and the IFG (mostly the frontal operculum), but also the most ventral part of DLPFC (Martino et al., 2010) by the AF white matter fibers. The superficial component of the dorsal route is underlain by the lateral part of the SLF III for articulatory processing. These cortical-subcortical positive sites are described by two articles: Herbet et al. (2018) for verbal semantic processing in the left hemisphere and for non-verbal semantics in the left and right hemispheres, and Zacà et al. (2018) for speech articulation. The cortical mapping results showed semantic paraphasia, phonological paraphasia, and speech arrest, respectively, during the intraoperative electrical stimulation of the right hemisphere. Nevertheless, these results were found in a much lower proportion in comparison with the data obtained in the left hemisphere. Of note, the FAT is also involved in speech initiation as well as in speech control (Dick et al., 2019). One article selected for the current systematic review (Kinoshita et al., 2014) is consistent with this fact. The authors describe FAT as a core of speech control due to the appearance of “speech initiation disturbances” during DES.

The ventral route, involving the IFOF, the inferior longitudinal fasciculus (ILF), and the uncinate fasciculus (UF), plays a role in verbal and non-verbal semantic processing. One article used in this systematic review (Herbet et al., 2017) agrees with these neuroanatomical findings within the right hemisphere. In this study, the authors found direct evidence for the role of the right IFOF in non-verbal semantic cognition, assessed by means of the PPTT. This finding confirms the homotypic organization of the non-verbal semantic network at the white matter level, consistent with the proposition made by Herbet et al. (2018) at the cortical level.

Language and speech disorders may arise post-operatively, emphasizing the need for language mapping in both hemispheres to prevent lasting impairments. The complexity of the brain's language network necessitates comprehensive assessment and mapping to ensure optimal outcomes.

### 4.2 Spatial cognition

Attention plays an important role in our daily lives, and the asymmetries of attentional networks that favor the right hemisphere (RH) are significant (Nobre, 2001; Corbetta and Shulman, 2002; Mandonnet and Herbet, 2021). Thus, dysfunction in these networks within the RH can lead to serious consequences. Impairments in attentional processes can impact many cognitive functions and behaviors, affecting an individual's ability to focus, perceive, and respond. Visual neglect is the dramatic clinical consequence of attention network dysfunction.

Spatial neglect or attentional disturbances are commonly assessed by several tests that can be broadly categorized into visuo-perceptual tests, visuo-graphic tests, and representational tests (Bartolomeo and Mandonnet, 2021). All these tasks were conducted in the articles reviewed in the present study. Even though it is remarkable that this cognitive domain is tested, sometimes with just one test (for example, only cancellation tasks), the line bisection task could be considered the gold standard test used to assess spatial cognition due to its level of evidence (100%). Consequently, this finding strongly reinforces the recommendation to include the line bisection task as part of the monitoring process during right parietal lobe surgeries.

Line bisection cortical positive sites seem to be highly variable from one patient to another, and when pooling all data from the literature, there is currently no more specific localization than the whole right parietal lobe (Bartolomeo and Mandonnet, 2021). Despite these findings, we found in the reviewed articles other positive cortical sites out from the parietal lobe, such as the precentral gyrus, the supramarginal gyrus, and motor areas. At the white matter level, electrostimulation studies have yielded consistent evidence suggesting the critical role of frontoparietal connections, supported by the SLF II and arcuate fasciculus pathways, in spatial awareness (Thiebaut de Schotten et al., 2005; Roux et al., 2011; Vallar et al., 2014). Several reports also observed a contribution from the IFOF (Herbet et al., 2017). These latter findings are consistent with the results obtained by the intraoperative stimulation of these white matter fibers performed in the spatial cognition articles selected for the present systematic review.

Other tasks beyond the line bisection task have been proposed as useful for mapping the networks involved in spatial attention, particularly cancellation-like tasks. While the line bisection task primarily evaluates the perceptual aspect of neglect, typically associated with parietal lesions, the cancellation task demands intentional exploration and attention orientation toward the contralesional space, a cognitive process rooted in the dorsal attention network (DAN), notably the eye field network. However, it is worth noting that a comprehensive lesion mapping study involving a cohort of patients with lower-grade glioma revealed that, despite resections targeting the medial eye field areas and their underlying connectivity or areas close to the frontal eye field, resulting in visuo-exploratory neglect, the deficit was transient (Herbet and Duffau, 2019). Since spatial cognition mapping with a cancellation task was not conducted in these areas, it raises questions about the additional utility of such tasks alongside the line bisection task.

### 4.3 Social cognition

Social cognition is thought to be supported by three anatomically separated but functionally interactive networks—the face perception network, the ‘mirror' neuron network, and mentalizing network (Keysers and Gazzola, 2007; Lieberman, 2007; Grill-Spector and Weiner, 2014; Pan et al., 2018; Wang et al., 2018). Mentalizing (or theory of mind) is fundamentally defined as the distinctively human tendency to attribute mental states (e.g., intentions, motives, and beliefs) to others based on observable behaviors and contextual information (Premack and Woodruff, 1978; Brothers, 1990). Mentalizing allows one to understand and anticipate others' behaviors and attitudes and to adjust one's own behavior in return.

Mentalizing is mainly assessed by the Read the Mind in the Eyes (RME; Baron-Cohen et al., 2001) test. The articles in the current systematic review relied heavily on this task, and they provided a high level of evidence (62.5%) supporting the importance of its use intraoperatively. RME should not be considered as a unique assessment of this cognitive function but rather a preferential gauge of social cognition.

The cortical areas involved in mentalizing-related processes include the dorsal and ventral aspects of the medial prefrontal cortex and the posterior cingulate cortex along with the precuneus (medially); the temporoparietal junction along with the posterior superior temporal sulcus, the IFG (laterally); and the temporal pole and its neighboring amygdala (less centrally) (Frith and Frith, 2003; Amodio and Frith, 2006; Carrington and Bailey, 2009; Van Overwalle and Baetens, 2009; Schurz et al., 2014). Likewise, it has been observed the important risk of generating long-term deficits in social cognition if there is a structural disconnection of the temporo-occipital areas and their connections via the ILF (Berro et al., 2021; Ekert et al., 2024). The white matter connections of the mentalizing system are, to date, understudied. However, a handle of studies has suggested that the white matter tracts that play a central role are the SLF/AF, the IFOF, and the cingulum (Herbet et al., 2014, 2016; Yordanova et al., 2017; Nakajima et al., 2018a,b; Roux et al., 2021). For example, Yordanova et al. (2017) explained that, cortically, the mapping results not only confirmed the involvement of the posterior part of the inferior frontal gyrus but also unveiled a pivotal role of the posterior dorsolateral prefrontal cortex, along with, to a lesser extent, the superior temporal gyrus (posterior part). Critically, numerous sites were also identified in the white matter underneath the dorsolateral prefrontal cortex and the middle and superior temporal gyri. The disconnection analyses performed on the axonal stimulation dataset demonstrated that fibers of the IFOF and the SLF/AF had the highest probabilities of being disconnected during subcortical stimulation. Additionally, Nakajima et al. (2018a,b), besides identifying positive cortical sites (IFG and SFG), found that not only the SLF/AF and IFOF are important in this process of social cognition. They found that FST and FAT have a significant contribution to specific aspects of mentalizing.

Some studies related to social cognition described deficits after the resection phase (Herbet et al., 2014; Yordanova et al., 2017; Roux et al., 2021). Although neuro-oncological studies suggest that surgical resections do not have a persistent detrimental effect on social cognition abilities, lesion mapping studies provide different results by showing degraded performances in social cognition abilities a few months after surgery. This discrepancy in results could be attributed to the fact that neuro-oncological studies typically do not employ a comprehensive array of neuroanatomical analyses (Nakajima et al., 2021).

### 4.4 Motor/sensory functions

Preventing motor impairments is one of the foremost considerations in brain tumor surgery, especially when the tumor is in proximity to the primary motor cortex and its associated descending nerve fibers. Consequently, cortical and subcortical brain mapping is often used to guide resection (Bello et al., 2021).

Tests for motor or sensory functions are less frequently mentioned. Many studies report that they monitor sensorimotor functions, but this is mostly done by asking a patient to report any sensations or movements (Rech et al., 2016, 2019; Motomura et al., 2018), and no specific task or paradigm is administered. Motor functions are also often monitored by motor-evoked potentials. Shinoura et al. (2010) and Rossi et al. (2018) describe the recently developed Hand Manipulation-Task (hMT), which requires the patient to perform a rotational movement on a tool shaped like a screwdriver. Many of the studies in this systematic review employed a dual task (75%), or in some of the articles, the neuropsychologist asked the patients to move the upper and lower limbs (50%). This renders these tasks the primary ones used in monitoring motor functions during right awake surgery. From a surgical viewpoint, specific patterns of positive or negative motor phenomena (both behavioral and EMG) are associated with different cortical-subcortical brain areas. Intraoperative DES applied over M1 causes negative responses. Additionally, the same negative responses were obtained after electrical stimulation of the dorsal premotor cortex (dPMC).

The SMA is linked to IFG and the ventral section of the precentral gyrus via the frontal aslant tract (FAT). Furthermore, the SMA, along with the dPMC and the ventral premotor cortex (vMPC), connects to the caudate nucleus through the fronto-striatal tract (FST) (Bello et al., 2021). The studies conducted by Kinoshita et al. (2014) and Rech et al. (2016) agree with these facts. In these articles, patients showed slight left or right paresis and “initiation disturbances” during DES applied over FAT and FST of the right hemisphere.

In most of the studies reviewed, the motor deficits were not permanent and tended to recover during the first 3 months (Shinoura et al., 2010; Kinoshita et al., 2014; Rech et al., 2016).

### 4.5 Executive functions

Executive functions (EFs), also known as executive control or cognitive control, encompass a set of top-down mental processes essential for concentration and attention, problem-solving, or behavioral control. There is general agreement that there are three core EFs (Miyake et al., 2000; Lehto et al., 2003): inhibition, working memory, and cognitive flexibility (also called set shifting).

The most studied EFs in the current systematic review are working memory and inhibitory control. Therefore, we will only describe the most significant findings related to these executive functions. In clinical settings, diverse tests address inhibitory mechanisms: go-on-go task or the stop-signal reaction-time task (Diamond, 2013; Motomura et al., 2018). However, the most used task is the N-back test. In the present systematic review, this task was employed to assess working memory in 57.14% of studies. The N-back test and the Stroop test have a good level of evidence. It could be interesting to add these tasks in the intraoperative protocol to evaluate working memory during right awake surgeries.

From a surgical viewpoint, intraoperative electrical stimulation has revealed some of the different networks sustaining these complex functions. In inhibitory control, the DLPFC seems to be crucial in the modulation of these processes. Motomura et al. (2018) obtained consistent results with this hypothesis in their study. In addition to this cortical region, they found the same disturbances during electrical stimulation of SFG. At the subcortical level, the right FAT could play a role in inhibitory control due to these white fibbers allowing the connection between the IFC and pre-SMA (Dick et al., 2019). Motomura et al. (2018) and Puglisi et al. (2019) agree with this fact. They found that the white matter direct stimulation highlighted the involvement of FAT in inhibitory control. Additionally, the involvement of SLF and AF in inhibitory control and cognitive flexibility seems to be clear. The study conducted by Hartung et al. (2021) confirmed that the right SLF and AF contributed to impairments in set-shifting and inhibition.

The current literature provides some evidence of long-lasting surgical deficits related to EFs. This fact indicates the necessity of assessing these high-order functions pre-, intra-, and post-operatively to avoid the high impact disturbances in the patient's quality of life.

### 4.6 Pre-, intra-, and post-operative neuropsychological assessment

The findings of the present systematic review underscore the critical importance of neuropsychological assessment before, during, and after awake craniotomy. Pre-operative evaluation establishes the patient's baseline neuropsychological profile, determining suitability for awake brain craniotomy and guiding intraoperative tasks (Hande et al., 2021). During surgery, neuropsychological tasks aid in directing the resection of brain tissue damage to optimize oncological outcomes (Kelm et al., 2017; Herbet and Duffau, 2020). The reviewed studies revealed resection percentages ranging from 70 to 100%, emphasizing the need to understand the networks involved in cognitive and emotional processing. Intraoperative neuropsychological assessments enable personalized resections within individual functional limits, fostering the development of functional neuro-oncology and connectome-based neurosurgery. Brain lesions can significantly impact functional connectivity, and disruptions in anatomical connectivity are well-documented (He et al., 2007; Griffis et al., 2019). The meta-networking approach, exploring how functional networks communicate and reconfigure in response to behavioral demands, offers a novel perspective (Herbet and Duffau, 2020). This approach may guide the selection of rehabilitation strategies to enhance between-network communication, leading to improved post-operative performance and a higher quality of life for patients.

### 4.7 Refining functional mapping of the right hemisphere

To refine the functional mapping of the right hemisphere during awake brain surgery, several strategies can be implemented to enhance the assessment of cognitive functions and improve the understanding of brain organization. Applying a comprehensive neuropsychological examination allows for a detailed evaluation of various cognitive domains, including visuospatial abilities, language processing, memory, social cognition, and sensory/motor functions (Strauss et al., 2006). By employing a wide range of standardized neuropsychological tests tailored to specific cognitive functions, clinicians can obtain a more nuanced understanding of the patient's cognitive profile and identify areas of potential functional impairment. Additionally, conducting longitudinal studies to assess functional recovery post-surgery can help identify which functions may not fully recover. Through the assessment of cognitive outcomes at various time intervals, researchers can ascertain the trajectory of recovery across different cognitive domains and obtain insights into the long-term effects of surgery on cognitive function. This longitudinal approach can inform treatment planning and rehabilitation strategies for patients with persistent cognitive deficits (Sala-Lonch et al., 2015; Gondar et al., 2021). Similarly, adapting tasks for mapping uncompensated functions in the right hemisphere is crucial for accurately identifying areas of cognitive vulnerability. Designing tasks that specifically target cognitive functions known to be challenging for the right hemisphere and incorporating sensitive measures of cognitive performance can effectively identify critical brain regions and assess functional integrity (Kelm et al., 2017; Herbet and Duffau, 2020). Tailoring tasks to probe specific cognitive processes enhances the accuracy of functional mapping and improves the identification of areas requiring preservation during surgery (Bates et al., 2003; Trimble et al., 2015). By incorporating these strategies into the assessment and mapping of right hemisphere functions during awake brain surgery, clinicians and researchers can refine their understanding of cognitive organization in the brain, heighten surgical outcomes, and enhance the quality of care for patients undergoing complex neurosurgical procedures.

### 4.8 Strengths and limitations of the study

The study exhibits notable strengths alongside certain limitations. It features a rigorous and comprehensive systematic search process, utilizing specific search terms and Boolean operators across multiple databases to identify relevant articles. This approach enhances the overall reliability of the literature review. Additionally, the study conducts in-depth analyses of selected articles, focusing on key variables related to cognitive and emotional functions during awake brain surgery, contributing to a thorough understanding of the research question. It also focuses on various cognitive functions, such as visuospatial abilities, language processing, memory, social cognition, and sensory/motor processes during awake brain surgery, allowing for a nuanced exploration of specific cognitive domains. Furthermore, it proposes a valuable protocol for assessing cognitive and emotional functions during awake brain surgeries, outlining six crucial tasks designed to evaluate different cognitive processes. This protocol can act as a foundational resource for guiding future research endeavors in this area. Moreover, the analysis of neural correlates associated with different cognitive functions assessed during awake brain surgery adds another layer of depth to the study, providing valuable insights into the underlying neural basis of cognitive processes within the unique context of brain surgery.

Despite these strengths, it is important to recognize the limitations of the study. Potential selection bias, which arises from a narrow focus on patient populations (adults with gliomas in the right hemisphere) who underwent awake craniotomies with direct electrical stimulation (DES), could restrict the applicability of the results to a wider population undergoing brain surgery. Methodological differences among the studies included variations in sample sizes, handedness, the use of diverse cognitive tasks, and the stimulation sites not specified in most of the selected articles, introducing complexities that could impact the comparability and integration of results. Additionally, inter-individual variability in plasticity potential, topographical bias due to neuroplasticity, and heterogeneity in glioma pathology (low-grade glioma vs. high-grade glioma) underscore the need for caution in interpreting results, considering individual differences, validating task sensitivity, addressing neuroplastic changes, and accounting for tumor characteristics. Understanding these limitations is essential for refining mapping techniques, optimizing patient outcomes, and advancing our understanding of brain plasticity in neurosurgical contexts.

In summary, considering both the advantages and drawbacks highlighted in this systematic review, clinicians and researchers can approach a more nuanced understanding of the insights regarding the right hemisphere awake brain surgery and its cognitive functions. This comprehension can then steer forthcoming research endeavors and enhancements in methodology within this domain.

## 5 Conclusions

This review examines visuospatial, language, memory, social cognition, and sensory/motor processes, along with corresponding neural structures, assessed during right awake craniotomy with direct electrical stimulation (DES) in adult glioma patients over the past 15 years. Analyzing high-impact journals, the present study challenges traditional views by revealing mirror brain circuits in the right hemisphere, emphasizing a distributed yet specialized network perspective. Dynamic interactions among these circuits lead to varied outcomes and neuroplastic phenomena, underscoring the importance of understanding the right hemisphere's functional anatomy in tumor surgery.

This systematic review proposes a protocol for assessing cognitive and emotional functions during right awake brain surgeries, identifying crucial tasks (DO80, PPTT, line bisection task, dual task, movements of the upper and lower limbs, RME, Stroop test, and N-back test) for language, spatial cognition, social cognition, motor/sensory functions, and executive functions. Implementing these tasks in a right hemisphere craniotomy protocol is deemed essential to prevent intraoperative injuries and post-operative effects, ensuring a comprehensive evaluation. These findings contribute to a better understanding of neuropsychological assessment within brain plasticity, emphasizing the need for careful task selection during intraoperative processes to prevent cognitive and emotional deficits and enhance patients' quality of life post-tumor resection.

## Data availability statement

The original contributions presented in the study are included in the article/supplementary material, further inquiries can be directed to the corresponding author.

## Author contributions

IM-M: Conceptualization, Formal analysis, Investigation, Supervision, Validation, Writing – original draft, Writing – review & editing. LA-C: Data curation, Formal analysis, Methodology, Supervision, Validation, Writing – original draft, Writing – review & editing. DS: Writing – review & editing. GH: Supervision, Writing – review & editing.
